# In Vitro Evaluation of the Efficacy of 27 French Over-the-Counter Anti-lice Treatments

**DOI:** 10.7759/cureus.86584

**Published:** 2025-06-23

**Authors:** Berthine Toubate, Aniss Louchez, Françoise Debierre, Thomas Morgenroth

**Affiliations:** 1 Infectiologie et Santé Publique (ISP), Université de Tours, Tours, FRA; 2 Pharmacy, Université de Lille, Lille, FRA

**Keywords:** clinical effectiveness, france, in vitro studies, pediculosis capitis, pediculosis humanus humanus

## Abstract

Head lice remain a major public health problem worldwide, with a high prevalence among schoolchildren. Many lice treatments are available, but their efficacy is often poorly evaluated. An increase in the resistance of parasites to neurotoxic therapies, combined with a partial efficacy of certain suffocating agents, can contribute to the maintenance or even rise in infestations. The study aims to evaluate the effectiveness of 27 different over-the-counter lice treatments available in France in 2024 for eliminating lice and nits. To do this, we used the subspecies Pediculus humanus humanus, a well-established model organism for competitive studies. The products were purchased in France from randomly selected pharmacies and then tested in the laboratory on lice and their eggs. The laboratory conditions of the in vitro tests were designed to reproduce the exposure times and application methods recommended by the manufacturers. A parallel study was carried out on sales data for anti-lice products from French pharmacies. The results allowed the classification of the 27 products into four groups: five products demonstrated potentially 100% pediculicidal and ovicidal activity; four products showed potentially 100% pediculicidal activity but insufficient ovicidal activity; another four products exhibited potentially 100% ovicidal activity but insufficient pediculicidal activity; and the remaining 14 products showed insufficient activity against both lice and nits. Sales data show that only 27% of products sold in French pharmacies are able to completely cure pediculosis. The French market for lice products is mainly occupied by treatments with a lack of effectiveness. Health authorities should require thorough efficacy assessments before registration and conduct periodic reviews to ensure the effectiveness of these treatments sold in pharmacies.

## Introduction

Head lice (*Pediculus humanus capitis*) are obligate blood-sucking parasites of humans, representing a worldwide public health problem. Indeed, it is estimated that 19% of school children worldwide are affected [1], with prevalence varying according to income level or gender [2].

The increase in prevalence was linked to the emergence of lice strains resistant to pediculicides with neurotoxic action [3-9], such as pyrethroids, which have long been the main source of treatment [10-19]. More recent manufacturers' strategies are based on physical action (suffocation of lice by blocking the respiratory stigmata) such as vegetable or mineral oils, silicone and its derivatives, or even a recent crystallizing agent. There is no uniform regulatory framework for these products, and their market authorization varies according to their mode of action. In France, while biocidal products are considered to be medicines, mechanically acting products are classified as class one medical devices, while other products may be considered cosmetics. Regulatory authorities should be encouraged to enforce their own rules to prevent this type of misleading terminology and advertising [20]. The lack of solid evidence for the efficacy of many topical head lice treatments makes it difficult for consumers and healthcare providers to make informed decisions, and often leads to a proliferation of insufficiently effective therapies.

The pediculosis market is well identified by the various players in the market, estimated in France at 70 million euros in 2019, 95% of which are pediculicide products [21]. The growth outlook, partly due to the opening of new markets in certain countries, also demonstrates the inability to limit the spread of lice and raises questions about the effectiveness of control methods.

To address this issue, manufacturers should be encouraged to provide explicit and more realistic packaging that is better aligned with the real application time and proper method of use, particularly if combing or reapplication is necessary. In pharmacies, patients can sometimes be misled by the instructions on the packaging, leaving them sometimes facing difficulties in applying treatment correctly, especially when dealing with very curly, frizzy, or long, thick hair, where combing becomes particularly challenging for certain hair types.

The first aim of the present study was to conduct an evaluation of the effectiveness of 27 lice treatments, available over-the-counter in pharmacies, in 2024 in France. For this, in vitro tests were performed on body lice (*Pediculus humanus humanus*) from a laboratory louse breeding, exhibiting similar reactions to head lice to treatments. The difficulty of maintaining the cycle of *Pediculus humanus capitis* in the laboratory justifies this choice of species, which also presents reactions similar to those of head lice to treatments. The second aim of the study was to investigate the financial burden of lice control in France.

## Materials and methods

Lice breeding and ethics statement

The colony of human body lice (*Pediculus humanus humanus*) was adapted to rabbit blood decades ago in the United States and maintained in laboratories around the world [22]. The laboratory-reared colony was kept at 30 ± 1 ˚C and 60%-70% relative humidity (Incu-Line, VWR International, Radnor, PA) without exposure to any drugs. Lice were fed four times per week for 20-30 minutes directly on rabbits (permit number of the French Ministry of Research APAFIS #27487-2020100713257288 v2).

Product application

Anti-lice products were purchased from random pharmacies by covert investigators and were categorized in four sub-categories based on their main components: (1) mineral oil, (2) vegetable oil, (3) silicone, and (4) crystallizing agent (Table 1).

**Table 1:** Identification and characteristics as indicated on the labeling of the 27 anti-lice products tested. MIPA, mono-isopropanolamine; DEA,diethanolamine

Product name (Company)	Galenic form	Principal component(s)	Other component(s)	Application time	Batch	Hair combing required
Mineral Oil						
Apaisyl® Anti-Poux Xpert (P&G Health France)	Lotion	Mineral oil (paraffinum liquidum)	Caprylic/capric glycerides PEG-8, propylene glycol monolaurate, PEG-6	1 hour	F1F12	Yes
Paranix® Express Rapide (Omega Pharma)	Lotion spray	Mineral oil (paraffinum liquidum	Isopropyl myristate, propylene glycol monolaurate, paraffinum liquidum, benzyl alcohol	2 minutes	B 4190431	Yes
Paranix® Extra Fort (New generation) (Omega Pharma)	Lotion	Mineral oil (Distillates (petroleum)	Sesamum indicum (sesame) seed oil, hydrotreated middle distillates, polyisobutene, isododecane, acrylates copolymer, geraniol, phenylethyl phenylacetate, citronellol, nerolidol isomers, geranyl acetate	5 minutes	L 1190421	Yes
Paranix® Shampoo Extra Fort (New generation) (Omega Pharma)	Shampoo	Mineral oil (paraffinum liquidum)	Laureth-4, MIPA-laureth sulfate, cocamide DEA, acrylates copolymer, isododecane, geraniol, phenylethyl phenylacetate, citronellol, nerolidol isomers, geranyl acetate, Sesamum indicum (sesame) seed oil	5 minutes	D 3251481	Yes
Vegetable Oil						
Apaisyl® Xpress (P&G Health France)	Lotion	Coco oil derivatives	Aqua, sodium methyl cocoyl taurate, propanediol, sodium laureth sulfate, PEG-8, citric acid, cocamidopropyl PG-dimonium chloride phosphate.	15 minutes	2 028	Yes
Balépou® Shampoo (Ineldea)	Shampoo	Coconut oil fatty acid (12%)	Aqua, sodium laureth sulfate, cocamidopropyl betaine, sodium chloride, parfum, tocopheryl acetate, chlorhexidine digluconate	15 minutes	231180B	Yes
Biogaran® Soin Traitant (Biogaran)	Lotion	Biocidine (Coconut fatty acid)	Preservative, fragrance, excipients. Paraben-free, phenoxyethanol-free. Silicone-free, insecticide-free	15 minutes	G 228659	Yes
Cinq/Cinq® Shampoo Gel (Bausch & Lomb)	Shampoo	Coco nucifera oil and cocomide MEA	Aqua, sodium laureth sulfate, cocamidopropyl betaine, tocopheryl acetate, methylchloroisothiazolinone, methylisothiazolinone, citric acid	15 minutes	C150	Yes
Duo LP Pro® (Omega Pharma)	Lotion	Oxyphthirine (film-forming agent based on triglycerides of natural origin)	Excipients QSP 200 mL	8 hours	D 32 81 011	Yes
Elimax® Shampoo (Oystershell)	Shampoo	LPF® (sesame oil, acrylate copolymer)	Hydrogenated didecene, MIPA-laureth sulfate, laureth-4, cocamide DEA, parfum	5 minutes	231 628	Yes
Expert 1.2.3® Lotion (Novodex)	Lotion	Coconut fatty acid	Aqua, potassium salts, glycerin, parfum	5 minutes	G C 10N0	Yes
Expert 1.2.3® Shampoo (Novodex)	Shampoo	Coconut fatty acid	Aqua, sodium laureth sulfate, cocamidopropyl betaine, sodium chloride, phenoxyethanol, citric acid, tocopheryl acetate, fragrance	20 minutes	G G 06N0	Yes
Parasidose® Lotion (Groupe Gilbert)	Lotion	LPF® (sesame oil, acrylate copolymer) and Prosil®	Aqua, glycerin, phenoxyethanol, benzyl alcohol, ethylhexylglycerin, parfum, xanthan gum	5 minutes	2023C23AB	Yes
Parasidose® Shampoo (Groupe Gilbert)	Shampoo	LPF® (sesame oil, acrylate copolymer)	Oligodecene oil, MIPA-laureth sulfate, laureth-4, cocamide DEA, fragrance	5 minutes	231 725	Yes
Polidis® Shampoo (Polidis)	Shampoo	Cocos Nucifera oil and Cocamide MEA	Aqua, sodium laureth sulfate, cocamidopropyl betaine, tocopheryl acetate, methylchloroisothiazolinone, methylisothiazolinone, citric acid	15 minutes	T0541	Yes
Pouxit Flash® Shampoo	Shampoo	Cocamide DEA	Isononyl isononanoate, MIPA-laureth sulfate, laureth-4, cocamide DEA	5 minutes	48RJ	Yes
Pouxit® Shampoo (Cooper)	Shampoo	Cocos Nucifera Oil and Cocamide MEA	Aqua, sodium trideceth sulfate, sodium lauroamphoacetate, sodium chloride, cocamidopropyl betaine, citric acid, hydroxypropyl guar, sodium benzoate, tetrasodium EDTA, potassium sorbate, tocopheryl acetate	15 minutes	C89	No
Puressentiel® Lotion (Puressentiel)	Lotion	Coco oil and essential oils	Tea tree essential oil, clove essential oil, true lavender essential oil, rose geranium essential oil	10 minutes	F 193272	Yes
Puressentiel® Shampoo Masque Traitant 2 en 1 (Puressentiel)	Shampoo	Coco oil	Unsweetened cocoa, sugar, cocoa butter, dietary fiber (inulin and oligofructose, dextrin), flax seeds (6%), puffed rice, emulsifier: soy lecithin (GMO free), natural vanilla. Cocoa solids: 65% minimum. Contains soy lecithin and gluten.	30 minutes	I 014045	Yes
Silicone Oil						
Cinq/Cinq® Lotion Baume (Bausch & Lomb)	Lotion	Dimethicone	Aqua, glycerine, Cocos Nucifera Oil, C12-15 alkyl benzoate, glyceryl stearate citrate, tocopheryl acetate, acrylates/C10-30 alkyl acrylate crosspolymer, Cera Alba, methylchloroisothiazolinone, methylisothiazolinone, sodium hydroxide	8 hours	C31	Yes
Expert 1.2.3® Lotion (Novodex)	Lotion spray	Dimethicone 4%	Cyclopentasiloxane 96%	15 minutes	28	Yes
Paranix® Lotion Extra Fort (Omega Pharma)	Lotion	Diméthicone	lyclol, dimethicone	10 minutes	E8190251	Yes
Polidis® Lotion Bi-Phase (Polidis)	Lotion spray	Dimethicone	aqua, peg-12, potassium sorbate, citric acid, melaleuca alternifolia, leaf oil.	15 minutes	C7	No
Pouxit® Flash (Cooper)	Lotion	Dimethicone (80% m/v)	Diisopropyl adipate, diisopropyl sebacate, lauryl alcohol, tocopheryl acetate	5 minutes	W22172F	Yes
Pouxit® XF (Cooper)	Lotion	Dimethicone	1,6,10-Dodecatrien-3-Ol, 3, 7, 11-Trimethyl, PEG/PPG Dimethicone Co-Polymer, Silica Silylate.	15 minutes	86RX	No
Viatris® DUO (Viatris)	Lotion	Dimethicone	Tocopherol acetate (vitamin E), apricot kernel oil, sweet almond oil.	15 minutes	341 565	Yes
Crystallizing agent						
Déparaz-Pro® (Duhot)	Lotion	Crystalysine® (oleoresine)	Acqua, Isoparaffin, laureth-4, Cocamide MIPA, Fatty acid ester, Citrus aurantium dulcis, Vitis Vinifera seed oil, pentylene glycol, Parfum limonene	15 minutes	CEB0093	No

The tests were carried out by following as closely as possible the manufacturer's instructions for use listed in the patient information leaflets, faithfully reproducing the application time and other applicable aspects of administration. For each tested product, 55 lice (3 batches of 18, 18, and 19, respectively) of different developmental stages (mix of L1, L2, and L3 larvae, and adults) or 51 to 145 viable nits (from different batches) laid out on tissue the day before, were used. They were placed in the lid of a 55 mm Petri dish (Lab-Online®) and then exposed to the product. In the case of lotion or shampoo indicated for application on dry hair, 400 μL of the product was poured onto the lice/nits. For shampoo indicated for application on wet hair, 100 μL of distilled water was first poured onto the lice/nits, followed by 400 μL of product. In the case of gel or balm textures, the product was applied with a spatula in sufficient quantity to completely cover the lice/nits. After 1 minute of complete immersion, product and lice/nits were transferred to the bottom of the Petri dish onto Whatman paper (Grade 1 CHR).

The contact between the product and the lice/nits was maintained for the exact time recommended by the manufacturer, at room temperature (19-23 °C), or 30 ± 1 °C and 50%-60% of relative humidity when the recommended application time exceeded 30 minutes.

After the set contact time, the lice/nits were washed on gauze with a children’s shampoo (Bébé Extra-Doux®, Repère®, Scamark®, Leclerc Group, Ivry-sur-Seine, France), non-toxic to lice and nits, diluted 1:3 in distilled water, and rinsed several times with distilled water until the last rinse became clear. Then, treated lice/nits were dried on a filter paper, transferred to a new Petri dish, with a new dry Whatman paper. Lice and nits were incubated at 30 ± 1 °C and 50%-60% relative humidity, for 24 hours and 10 days, respectively. As a control, 55 lice or 59 nits were subjected to the same procedures with distilled water. Several products were evaluated simultaneously with a single control batch.

Monitoring pediculicidal and ovicidal activities

Lice were examined under a stereo microscope (Motic DM143 series) 5 minutes, 15 minutes, 30 minutes, 1 hour, and 24 hours after the final rinse of the washing step. The physical status of lice post-treatment was classified into three categories: (1) alive, indicating normal behavior and movement; (2) knockdown (KD), defined by ataxia affecting the legs, antennae, and digestive tract; and (3) dead, characterized by a complete absence of biological activity. Given that most product claims are based on a 30-minute action time, results at this time point were emphasized and compared to those observed after a 24-hour exposure.

Nits were observed under a stereo microscope daily for 10 days and categorized into two classes: alive (hatched) and dead (non-hatched or dead during the hatching process). The primary endpoint was the percentage of mortality at day 10.

Statistical analysis

The minimum sample size of 55 lice per experimental group was chosen to ensure sufficient power to assess with high precision the true prevalence of lice killed by the test products and to determine with a 95% confidence interval whether the efficacy in the treated group is different from 100% or not. A Student's t-test was performed under the null hypothesis of 100% effectiveness using the statistical software R. Given the observed proportions of dead lice were close to 100%, exact 95% confidence intervals were also calculated.

Financial impact study

Sales data for anti-lice products were acquired from a software publisher covering 10,321 pharmacies out of the 19,887 listed in France [23], for the period April 2023 to March 2024. The characteristics and location of pharmacies were not taken into account. A total of 401 product listings were identified, most of which were variations of the same product offered in different volumes. For example, Apaisyl XPERT® is available in six variants (100-300 mL), including one with a magnifying glass. Sales data for these products were aggregated, and a weighted average selling price was calculated based on quantities sold. Only products available on the French market were included in the analysis. One of the 27 tested products, Elimax Shampoo®, was not sold by any of the pharmacies in the data sample and was only available for purchase online. The remaining 26 products represented 101 variations (galenic formulations, volumes, and presentations) and accounted for 87.35% of the total pharmacy sales of all products (including repellents) in the data sample.

## Results

**Figure 1:**
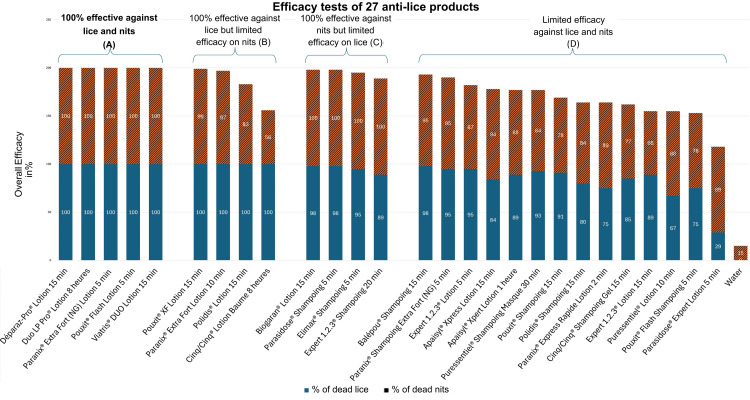
Tests of 27 anti-lice products. The figure presents product efficacy grouped into four categories based on their effectiveness profiles: (A) 100% effective against both lice and nits, (B) 100% effective against lice but limited efficacy on nits, (C) 100% effective against nits but limited efficacy on lice, and (D) limited efficacy on both lice and nits. Efficacy is assessed by the percentage of mortality, with lice represented in blue and nits in red. On the y-axis, a value of 200% indicates complete mortality of both lice and nits (Group A, shown in bold).

**Figure 2:**
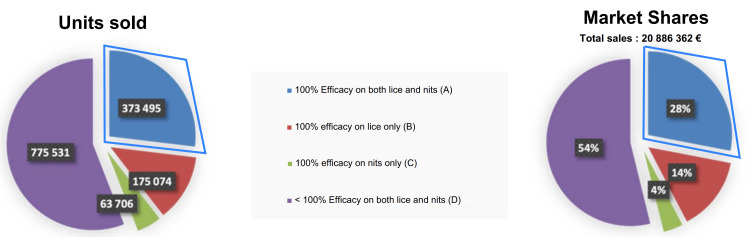
Units sold and market shares in France for 26 pediculicides (April 2023-March 2024). This figure presents the market share of anti-lice products by category, shown in units sold (left graph) and as percentages (right graph). Category A, which demonstrates 100% efficacy against both lice and nits, is highlighted in blue. Categories B, C, and D are represented in red, green, and purple, respectively.

The pediculicidal and ovicidal activities of the 27 tested products and the control groups are presented in Tables 2-3 and illustrated in Figure 1. Based on the raw data, 27 products were classified into four groups, according to their efficacy. The results were ranked by product effectiveness, and when products had identical efficacy, they were ranked by alphabetical order (Figure 1). Sales data for 26 of these products are presented in Figure 2.

**Table 2:** Mortality of lice (pediculicidal activity) after one in vitro exposure This table presents lice mortality for each product at 30 minutes and 1 hour. Each cell is formatted to show the percentage of lice mortality ± standard deviation (n = number of lice). Product names in bold indicate those with 100% efficacy against both lice and nits (Category A).

Agents with physical action	Products	Experimental group	Time after contact and washing					
			After 30 minutes			After 24 hours		
		N	% dead +/- 95% CI (*n*)	% knockout +/- 95% CI (*n*)	% alive +/- 95% CI (*n*)	% dead +/- 95% CI (*n*)	% knockout +/- 95% CI (*n*)	% alive +/- 95% CI (*n*)
Mineral oils	Apaisyl® Xpert Lotion 1 hour	55	0 +/-0 (*n *= 0)	100 +/-0.58 (*n *= 55)	0 +/-0 (*n *= 0)	89.09 +/-0.58 (*n *= (*n *= 49)	0 +/-0 (*n *= 0)	10.91 +/- 1.00 (*n *= 6)
	Paranix® Express Rapide Lotion 2 minutes	55	0 +/-0 (*n *= 0)	81.82 +/- 2.00 (*n *= 45)	18.18 +/- 1.53 (*n *= (*n *= (*n *= 10)	74.55 +/- 1.53 (*n *= 41)	0 +/-0 (*n *= 0)	25.45 +/- 1.00 (*n *= 14)
	Paranix® Extra Fort (NG) Lotion 5 min	55	0 +/-0 (*n *= 0)	100 +/-0.58 (*n *= 55)	0 +/-0 (*n *= 0)	100 +/-0.58 (*n *= 55)	0 +/-0 (*n *= 0)	0 +/-0 (*n *= 0)
	Paranix® Shampoo Extra Fort (NG) 5 min	55	0 +/-0 (*n *= 0)	94.55 +/-0.58 (*n *= 52)	5.45 +/- 1.00 (*n *= 3)	94.55 +/-0.58 (*n *= 52)	0 +/-0 (*n *= 0)	5.45 +/- 1.00 (*n *= 3)
Vegetable oils	Apaisyl® Xpress Lotion 15 min	55	0 +/-0 (*n *= 0)	94.55 +/-0.58 (*n *= 52)	5.45 +/- 1.00 (*n *= 3)	83.64 +/- 1.15 (*n *= 46)	0 +/-0 (*n *= 0)	16.36 +/- 1.53 (*n *= 9)
	Balépou® Shampoo 15 min	55	0 +/-0 (*n *= 0)	100 +/-0.58 (*n *= 55)	0 +/-0 (*n *= 0)	98.18 +/- 2.00 (*n *= 54)	0 +/-0 (*n *= 0)	1.82 +/- 0.58 (*n *= 1)
	Biogaran® Lotion 15 min	55	0 +/-0 (*n *= 0)	98.18 +/- 2.00 (*n *= 54)	1.82 +/- 0.58 (*n *= 1)	98.18 +/- 2.00 (*n *= 54)	0 +/-0 (*n *= 0)	1.82 +/- 0.58 (*n *= 1)
	Cinq/Cinq® Shampoo Gel 15 min	55	0 +/-0 (*n *= 0)	100 +/-0.58 (*n *= 55)	0 +/-0 (*n *= 0)	85.45 +/- 1.00 (*n *= 47)	0 +/-0 (*n *= 0)	14.55 +/- 0.58 (*n *= 8)
	Duo LP Pro® Lotion 8 hours	55	0 +/-0 (*n *= 0)	100 +/-0.58 (*n *= 55)	0 +/-0 (*n *= 0)	100 +/-0.58 (*n *= 55)	0 +/-0 (*n *= 0)	0 +/-0 (*n *= 0)
	Elimax® Shampoo 5 min	55	0 +/-0 (*n *= 0)	100 +/-0.58 (*n *= 55)	0 +/-0 (*n *= 0)	94.55 +/-0.58 (*n *= 52)	0 +/-0 (*n *= 0)	5.45 +/- 1.00 (*n *= 3)
	Expert 1.2.3® Lotion 5 min	55	0 +/-0 (*n *= 0)	100 +/-0.58 (*n *= 55)	0 +/-0 (*n *= 0)	94.55 +/-0.58 (*n *= 52)	0 +/-0 (*n *= 0)	5.45 +/- 1.00 (*n *= 3)
	Expert 1.2.3® Shampoo 20 min	55	0 +/-0 (*n *= 0)	100 +/-0.58 (*n *= 55)	0 +/-0 (*n *= 0)	89.09 +/-0.00 (*n *= 49)	0 +/-0 (*n *= 0)	10.90 +/- 0.58 (*n *= 6)
	Parasidose® Expert Lotion 5 min	55	0 +/-0 (*n *= 0)	72.73 +/- 2.31 (*n *= 40)	27.27 +/- 2.65 (*n *= 15)	29.09 +/-4.36 (*n *= 16)	0 +/-0 (*n *= 0)	70.91 +/- 4.62 (*n *= 39)
	Parasidose® Shampoo 5 min	55	0 +/-0 (*n *= 0)	98.18 +/- 2.00 (*n *= 54)	1.82 +/- 0.58 (*n *= 1)	98.18 +/- 2.00 (*n *= 54)	0 +/-0 (*n *= 0)	1.82 +/- 0.58 (*n *= 1)
	Polidis® Shampoo 15 min	55	0 +/-0 (*n *= 0)	100 +/-0.58 (*n *= 55)	0 +/-0 (*n *= 0)	80.00 +/- 0.58 (*n *= 44)	0 +/-0 (*n *= 0)	20.00 +/- 0.00 (*n *= 11)
	Pouxit® Flash Shampoo 5 min	55	0 +/-0 (*n *= 0)	90.91 +/- 1.15 (*n *= 50)	9.09 +/- 0.58 (*n *= 5)	74.55 +/-0.58 (*n *= 41)	0 +/-0 (*n *= 0)	25.45 +/-0.58 (*n *= 14)
	Pouxit® Shampoo 15 min	55	0 +/-0 (*n *= 0)	100 +/-0.58 (*n *= 55)	0 +/-0 (*n *= 0)	90.91 +/- 2.65 (*n *= 50)	0 +/-0 (*n *= 0)	9.09 +/- 2.08 (*n *= 5)
	Puressentiel® Lotion 10 min	55	0 +/-0 (*n *= 0)	87.27 +/- 1.00 (*n *= 48)	12.73 +/- 0.58 (*n *= 7)	67.27 +/- 1.53 (*n *= 37)	0 +/-0 (*n *= 0)	32.73 +/- 1.00 (*n *= 18)
	Puressentiel® Shampoo Masque 30 min	55	0 +/-0 (*n *= 0)	100 +/-0.58 (*n *= 55)	0 +/-0 (*n *= 0)	92.73 +/- 4.04 (*n *= 51)	0 +/-0 (*n *= 0)	7.27 +/- 1.00 (*n *= 4)
Silicone	Cinq/Cinq® Lotion Baume 8 hours	55	0 +/-0 (*n *= 0)	100 +/-0.58 (*n *= 55)	0 +/-0 (*n *= 0)	100 +/-0.58 (*n *= 55)	0 +/-0 (*n *= 0)	0 +/-0 (*n *= 0)
	Expert 1.2.3® Lotion 15 min	55	0 +/-0 (*n *= 0)	100 +/-0.58 (*n *= 55)	0 +/-0 (*n *= 0)	89.09 +/- 1.53 (*n *= 49)	0 +/-0 (*n *= 0)	10.91 +/- 1.00 (*n *= 6)
	Paranix® Extra Fort 10 min	55	0 +/-0 (*n *= 0)	100 +/-0.58 (*n *= 55)	0 +/-0 (*n *= 0)	100 +/-0.58 (*n *= 55)	0 +/-0 (*n *= 0)	0 +/-0 (*n *= 0)
	Polidis® Lotion 15 min	55	0 +/-0 (*n *= 0)	100 +/-0.58 (*n *= (*n *= 55)	0 +/-0 (*n *= 0)	100 +/-0.58 (*n *= (*n *= 55)	0 +/-0 (*n *= 0)	0 +/-0 (*n *= 0)
	Pouxit® Flash 5 min	55	0 +/-0 (*n *= 0)	100 +/-0.58 (*n *= 55)	0 +/-0 (*n *= 0)	100 +/-0.58 (*n *= 55)	0 +/-0 (*n *= 0)	0 +/-0 (*n *= 0)
	Pouxit® XF Lotion 15 min	55	0 +/-0 (*n *= 0)	100 +/-0.58 (*n *= 55)	0 +/-0 (*n *= 0)	100 +/-0.58 (*n *= 55)	0 +/-0 (*n *= 0)	0 +/-0 (*n *= 0)
	Viatris® DUO Lotion 15 min	55	0 +/-0 (*n *= 0)	100 +/-0.58 (*n *= 55)	0 +/-0 (*n *= 0)	100 +/-0.58 (*n *= 55)	0 +/-0 (*n *= 0)	0 +/-0 (*n *= 0)
Crystallizing agent	Déparaz Pro® Lotion 15 min	55	0 +/-0 (*n *= 0)	100 +/-0.58 (*n *= 55)	0 +/-0 (*n *= 0)	100 +/-0.58 (*n *= 55)	0 +/-0 (*n *= 0)	0 +/-0 (*n *= 0)
Control group	Water	55	0 +/-0 (*n *= 0)	0 +/-0 (*n *= 0)	100 +/-0.58 (*n *= 55)	0 +/-0 (*n *= 0)	0 +/-0 (*n *= 0)	100 +/-0.58 (*n *= 55)

**Table 3:** Mortality of nits (ovicidal activity) after one in vitro exposure.

Agents with physical action	Products	Experimental group	10 days after contact and washing
		*N*	% dead (*n*)
Mineral oils	Apaisyl® Xpert Lotion 1 heure	145	88 (*n* = 128)
	Paranix® Express Rapide Lotion 2 min	53	89 (*n* = 47)
	Paranix® Extra Fort (NG) Lotion 5 min	56	100 (*n* = 56)
	Paranix® Shampoo Extra Fort (NG) 5 min	51	95 (*n* = 48)
Vegetable oils	Apaisyl® Xpress Lotion 15 min	52	94 (*n* = 49)
	Balépou® Shampoo 15 min	59	95 (*n* = 56)
	Biogaran® Lotion 15 min	71	100 (*n* = 71)
	Cinq/Cinq® Shampoo Gel 15 min	57	77 (*n* = 44)
	Duo LP Pro® Lotion 8 heures	52	100 (*n* = 52)
	Elimax® Shampoo 5 min	61	100 (*n* = 61)
	Expert 1.2.3® Lotion 5 min	53	87 (*n* = 46)
	Expert 1.2.3® Shampoo 20 min	103	100 (*n* = 103)
	Parasidose® Expert Lotion 5 min	83	89 (*n* = 74)
	Parasidose® Shampoo 5 min	53	100 (*n* = 53)
	Polidis® Shampoo 15 min	55	84 (*n* = 46)
	Pouxit® Flash Shampoo 5 min	94	78 (*n* = 73)
	Pouxit® Shampoo 15 min	74	78 (*n* = 58)
	Puressentiel® Lotion 10 min	57	88 (*n* = 50)
	Puressentiel® Shampoo Masque 30 min	68	84 (*n* = 57)
Silicone	Cinq/Cinq® Lotion Baume 8 heures	87	56 (*n* = 49)
	Expert 1.2.3® Lotion 15 min	89	66 (*n* = 59)
	Paranix® Lotion Extra Fort 10 min	115	97 (*n* = 112)
	Polidis® Lotion 15 min	75	83 (*n* = 62)
	Pouxit® Flash Lotion 5 min	64	100 (*n* = 64)
	Pouxit® XF Lotion 15 min	89	99 (*n* = 88)
	Viatris® DUO Lotion 15 min	97	100 (*n* = 97)
Crystallizing agent	Déparaz Pro® Lotion 15 min	55	100 (*n* = 55)
Control group	Water	59	15 (*n* = 9)

The 27 products were classified into four groups according to their effectiveness: a first group of five products with potentially 100% pediculicidal activity (killing 100% of the mobile stage of lice) and potentially 100% ovicidal activity (killing 100% of the viable nits); a second group of four products with potentially 100% pediculicidal activity and insufficient ovicidal activity; a third group of four products with potentially 100% ovicidal activity and insufficient pediculicidal activity; and a final group of 14 products with insufficient pediculicidal and ovicidal activity.

In the first group, the five products with 100% efficacy against both lice and nits have distinct principal ingredients: a crystallizing agent (Déparaz-Pro® Lotion 15 minutes), vegetable oil (Duo LP Pro® Lotion 8 hours), mineral oil (Paranix® Extra Fort (NG) Lotion 5 minutes), or silicone (Pouxit® Flash Lotion 5 minutes, Viatris® DUO Lotion 15 minutes). Despite the high efficacy of Pouxit XF Lotion 15 minutes, this product was not included; therefore, this category contains only products that did not differ in efficacy within our study.

This product category accounted for approximately 27% of the market share in units sold (Figure 2). Notably, one product containing silicone was well ahead of the others, representing just over 62% of the sales within this category.

The second group included four products that were 100% effective against lice but showed limited efficacy on nits. All of these products contained silicone (Paranix® Lotion Extra Fort 10 minutes, Polidis® Lotion Bi-Phase 15 minutes, Pouxit® XF Lotion 15 minutes, Cinq/Cinq® Lotion Baume 8 hours). While this group of products accounted for around 13% of the market share, it is noteworthy that one of these products accounted for 89% of the sales within the group (Figure 2).

The third group consisted of four products that were 100% effective against nits but had limited efficacy against lice. It included three shampoos (Elimax® Shampoo 5 minutes, Parasidose® Shampoo 5 minutes, Expert 1.2.3® Shampoo 20 minutes) and one lotion (Biogaran® Soin Traitant Lotion 15 minutes), all of which contained either mineral or vegetable oils. This category represented only 5% of the market share of products sold in our data sample (Figure 2). However, one of these products was not included in the sample, as it was sold exclusively online.

The last group was made up of 14 products showing limited efficacy against both lice and nits, and represented around 55% of total sales in our data sample (Figure 2). Surprisingly, this category constituted the main group of products, with a variety of formulations. They contained either vegetable oils (Expert 1.2.3® Lotion 5 minutes, Parasidose® Expert Lotion 5 minutes, Pouxit® Flash Shampoo 5 minutes, Puressentiel® Lotion 10 minutes, Apaisyl® Xpress Lotion 15 minutes, Balépou® Shampoo 15 minutes, Cinq/Cinq® Shampoo 15 minutes, Polidis® Shampoo 15 minutes, Pouxit® Shampoo 15 minutes, Puressentiel® Shampoo Mask 30 minutes), mineral oils (Paranix® Express Rapide Lotion 2 minutes, Paranix® Shampoo Extra Fort (NG) 5 minutes, Apaisyl® Xpert Lotion 1 hour), or silicone (Expert 1.2.3® Lotion 15 minutes).

## Discussion

The efficacy of certain pediculicides has been the subject of previous publications [24-30]. Recent studies have focused on a comparison of a limited number of products [27,31]. Some studies have tested a wider range of products using artificial lice feeding methods [32] or by ex vivo tests on head lice collected from children [33]. Other studies conducted in Egypt [34] and the United States [6,35] have tested anti-lice products; however, they did not follow the manufacturer’s instructions or evaluate ovicidal efficacy.

The evolution of the composition of pediculicide products, as well as the modes of action, implies an update of their efficacy test. However, in clinical studies, biases may arise in the method of using the treatment, particularly when combining is required. In this case, the resulting efficacy cannot be directly linked to the product itself.

Our study reveals that the five products with 100% effectiveness against both lice and nits act through physical mechanisms - suffocation in four cases and crystallization in one. The mode of action by suffocation gives variable results, as the other products tested that adopt the same mechanism do not achieve similar scores. This could be due to the percentage of the active agent and/or to the role of the other components in the formula. The crystallization process seems to stand out for its effectiveness. However, as only one product of this type is marketed (Déparaz-Pro®), no comparison is possible. It is also noteworthy that no shampoo achieved complete eradication of both lice and nits.

The results of this study highlight a number of concerns about the effectiveness of lice treatments currently available on the French market. We can consider that the products tested, which are not 100% effective on lice and nits, may not be capable of curing pediculosis, since a single live gravid female or two to three viable eggs can give rise to a new infesting generation.

This study questions manufacturers’ marketing strategies, as the efficacy rates displayed on packaging can mislead the user [36]. Some products recommend a second application, which can lead to doubts about their efficacy. Consumers expect a product to be fully effective after the first application, as often shown on packaging, not after multiple repeated uses after 7-10 days. Application methods also vary greatly from product to product and are not always clearly indicated on the packaging or instructions. First, the claims on the packaging of certain products are misleading, particularly regarding application times. Some packaging promises a cure in a few minutes, but these treatments are often associated with combing or need to be reapplied at day 7 or multiple cleansing shampoos. Furthermore, the study results show efficacy times that are sometimes longer than those stated on the packaging, arguing for times that should be extended. Indeed, the combing - a laborious task, which considerably increases the treatment time, varying according to the nature of the hair. And some products are designed to be used as a combing aid (i.e., have no or low intrinsic activity to kill lice or their eggs), and this should be indicated when this is the case. In addition, combing treatment may not be applicable for very curly, frizzy, or long, thick hair.

This situation raises important questions in terms of public health. The predominance of ineffective or partially effective products, which represent 72% of the units sold in our sample of pharmacies, probably contributes to maintaining contamination and parasite spreading. Furthermore, certain treatments, such as those based on highly concentrated silicones (dimethicone), may be concerning [37,38]. In particular, silicones like D4 (octamethylcyclotetrasiloxane) and D5 (decamethylcyclopentasiloxane) are known environmental pollutants. They persist in the ecosystem and accumulate in aquatic environments, contributing to long-term ecological damage. In addition, research has indicated that D4 may have adverse effects on human health, including potential impacts on fertility [39], raising further concerns about the widespread use of highly concentrated silicones in lice treatments.

It could be noticed that among the products effective against lice, the products with the highest sales are those containing silicone, representing 69% of sales of products 100% effective on lice and nits, and 100% of sales of products effective only against lice, respectively. For better pharmacy advice, the focus should shift towards recommending products with proven efficacy, easy to use, cosmetics-friendly, and, if possible, avoiding polluting ingredients. This would help patients to make more informed decisions and improve the success of treatments against lice in all hair types and conditions. It can be noted that among the effective products against lice, the best-selling products are those containing silicone.

Limitations

The success of the in vitro method proposed here should be further validated by a randomized controlled clinical trial.

It is also possible that the treatment under these experimental conditions, with optimal immersion of lice and nits in relatively large quantities of anti-lice product and full compliance with exposure times, is more effective than in real application. For example, some users comb their hair immediately after applying the product, instead of waiting the recommended time, thus removing a large part of it. Thus, further investigations under real-life conditions are needed to account for practical variability, such as combing, and quantify the environmental effects associated with silicones.

On the other hand, the combing process, recommended by some manufacturers to remove dead parasites, in addition to the application of the product, can influence the final efficacy [40], by removing lice and eggs that might still be alive. It is, therefore, more likely that the least effective products are rather combining adjuvants, acting as a lubricant for the hair, and just facilitating combing.

The selection of products is based on those available in France in 2024 and excludes the study of certain neurotoxic products, which could be used in other countries of the world. The French health authority had reclassified a product (Prioderm Lotion®) containing malathion [41] and terpineol, considering the risks of causing neurological disorders at excessive doses. As the product is subject to mandatory prescription, the manufacturer preferred to withdraw the product from the market.

In addition, the financial study cannot accurately reflect the market for anti-lice products, as it is based on only part of the pharmaceutical sales network, which is in competition with other distribution networks, such as supermarkets or the web. Nevertheless, consumers are entitled to expect better advice from a health professional, incorporating the real and proven effectiveness of products.

The evolving nature of the products, particularly due to commercial considerations, may lead in the short term to the products studied no longer being marketed. Nevertheless, despite the change in the name of the product, the mode of action and composition could remain close to the existing products.

## Conclusions

Among the 27 tested products sold in pharmacies, only nine were able to kill lice, and only five of those also eliminated nits. Although these results were obtained using body lice, they are considered representative of efficacy against head lice. Thus, the French market for lice treatments is largely saturated with products that fail to achieve full efficacy. To safeguard public health, authorities should enforce rigorous efficacy evaluations before approving new treatments and conduct regular reviews to ensure that pharmacy-sold products continue to meet high standards of effectiveness.
